# Eddy Covariance vs. Reduced-Aperture Scintillometry for Potato Crop Evapotranspiration in the Beqaa Valley, Lebanon

**DOI:** 10.3390/s26144398

**Published:** 2026-07-10

**Authors:** George Rahhal, Hadi Jaafar

**Affiliations:** Department of Agriculture, Faculty of Agricultural and Food Sciences, American University of Beirut, Beirut 1107 2020, Lebanon

**Keywords:** evapotranspiration, eddy covariance, scintillometry, energy-balance closure, Bowen ratio, potato, irrigation, MENA, Beqaa Valley

## Abstract

Accurate estimation of evapotranspiration (ET) is critical for irrigation management in water-scarce regions such as the Middle East and North Africa (MENA). This study compares sensible heat flux (H), latent heat flux (LE), and ET derived from eddy covariance (EC) and a boundary-layer scintillometer (BLS) operated with an aperture reducer, deployed simultaneously over an irrigated late-season potato field (1.8 ha) in the Beqaa Valley, Lebanon. Satellite NDVI observations indicate that the BLS–EC overlap period (13 October–27 November 2021) sampled the crop from peak canopy (NDVI ≈ 0.85–0.90) through the onset of senescence (NDVI ≈ 0.79). The BLS (Scintec BLS900) operated along a 140 m path. The EC system showed incomplete daytime energy-balance closure, with a regression slope of ≈0.69 and a seasonal Bowen-ratio-preserving correction factor of CF = 1.24 (a ~19% closure deficit) was used. Across the matched period, daily H from the BLS was strongly correlated with EC (r ≈ 0.82) but systematically lower, with a regression slope of ≈0.63 that persisted across timescales; this scale-invariant amplitude compression reflects the path-averaged, similarity-based nature of the scintillometer retrieval rather than the EC closure deficit, which instead governs the mean bias. BLS-derived daily ET showed a systematic positive bias relative to uncorrected EC (mean bias error, MBE = +0.30 mm d^−1^; +16% cumulative). Applying the Bowen-ratio-preserving correction (CF = 1.24) to EC reduced this to MBE = −0.14 mm d^−1^ (−6%), and the residual-to-LE correction yielded MBE = −0.15 mm d^−1^ (−6.4%); the latter comparison is only partly independent, as both methods share the same Rn and G. The Bowen-ratio-preserving method is therefore recommended for this dataset. Overall, the BLS captured the temporal variability of crop water use well, but residual-based ET estimates require careful treatment of the energy-balance-closure gap and are sensitive to the high BLS gap fraction (61.6% of 15 min records over the overlap, exceeding 90% at night). Once EC is closure-corrected to serve as the reference, the BLS offers a cost-effective alternative for field-scale ET monitoring in the MENA region, subject to the conditional agreement documented here.

## 1. Introduction

Agriculture in the Middle East and North Africa (MENA) is under growing pressure from severe droughts and competing demands for limited water resources [1]. The intensifying water crisis threatens crop productivity and food security, highlighting the need for efficient irrigation management and accurate estimation of evapotranspiration (ET), a key variable in determining crop water requirements and irrigation scheduling [1]. Evapotranspiration governs critical ecosystem processes including soil moisture dynamics, vegetation productivity, nutrient cycling, and catchment water budgets [2]. As rising temperatures enhance the atmosphere’s moisture-holding capacity and intensify the hydrological cycle [3], accurate ET estimation becomes increasingly important for sustainable water management.

Several micrometeorological methods are available for measuring ET, including the eddy-covariance (EC) technique and large-aperture scintillometry. The EC technique is widely regarded as the reference standard for surface energy flux measurements [4]. It directly measures turbulent fluxes of sensible heat, latent heat, and momentum from high-frequency covariance between vertical wind velocity and scalar fluctuations, using a three-dimensional sonic anemometer and fast-response gas analyzer [5]. EC has been applied extensively across diverse ecosystems, including croplands [6], forests [7], grasslands [2], and wetlands [8,9]. However, EC systems are costly, require expert maintenance, and often exhibit incomplete energy-balance closure—a non-closure of 10–30% is commonly reported across agricultural FLUXNET sites [10,11,12]. This non-closure can introduce significant uncertainty in daily ET estimates—sometimes up to 50%—particularly over heterogeneous or advective agricultural surfaces [6,13]. The energy imbalance complicates the use of EC as an absolute benchmark for evaluating other flux measurement techniques, as it remains unclear how much of the discrepancy is due to sensor error versus uncaptured low-frequency eddies or footprint mismatches [14].

Scintillometry determines sensible heat flux from the refractive-index structure parameter of air (C_n_^2^), measured via fluctuations in the intensity of an electromagnetic beam [15,16]. Scintillometry provides path-averaged fluxes over scales of hundreds of meters to kilometers, offering better spatial representativeness than point measurements [17]. However, conventional large-aperture scintillometers (LAS) require long, homogeneous fetches and extensive, uniform surfaces to satisfy footprint and Monin–Obukhov similarity theory (MOST) assumptions. In heterogeneous agricultural landscapes, such long path lengths may integrate fluxes across multiple crop types or land covers, limiting the ability to attribute ET to a specific field. To address this, reduced-aperture scintillometer (BLS) systems with shorter path lengths can be deployed over individual fields, enabling field-scale flux estimation while maintaining path-averaged robustness. Nevertheless, scintillometers rely on MOST and can be sensitive to humidity fluctuations (Bowen-ratio effects) and saturation of the scintillation signal under strong turbulence [18,19]. A persistent challenge in using BLS as a residual-based ET method is the spatial footprint mismatch: while sensible heat is path-averaged, the available energy components are typically measured at a point scale, which can introduce discrepancies in heterogeneous irrigated fields [20,21].

Despite EC being the de facto reference for surface flux measurement, its persistent energy-balance closure deficit complicates its use as an absolute benchmark—precisely the difficulty when evaluating a residual-based method such as BLS, whose latent heat flux is obtained as LE = Rn − G − H and therefore inherits any error in the available energy term (Rn − G). This evaluation is most demanding over heavily irrigated, fully transpiring surfaces, where the Bowen ratio (β = H/LE) fluctuates near and below zero: with H small relative to LE, residual LE becomes highly sensitive to errors in (Rn − H − G), while the small and occasionally negative H simultaneously challenges the BLS humidity correction. Simultaneous, co-located BLS–EC comparisons under this regime—quantifying how the EC closure deficit propagates into residual-based BLS LE over a negative- to near-zero-β, peak-canopy irrigated crop—have not, to our knowledge, been reported for the MENA region.

Accordingly, this study presents an intercomparison of EC and reduced-aperture scintillometry (BLS) for estimating surface energy fluxes and ET over an irrigated potato field in the Beqaa Valley of Lebanon. The specific objectives are: (i) to compare sensible and latent heat fluxes measured by EC and BLS at 30 min, hourly, daily, and weekly scales, using crop phenology to contextualize the observed turbulent fluxes; (ii) to quantify the impact of energy-balance-closure corrections (Bowen-ratio-preserving, [22]; and residual-to-LE, [23]) on the discrepancy between residual-based (BLS) and direct (EC) ET estimates; and (iii) to evaluate the applicability of these methods for irrigation management in the MENA region, with explicit acknowledgement of the conditional nature of the agreement.

## 2. Materials and Methods

### 2.1. Site Description

**Figure 1:**
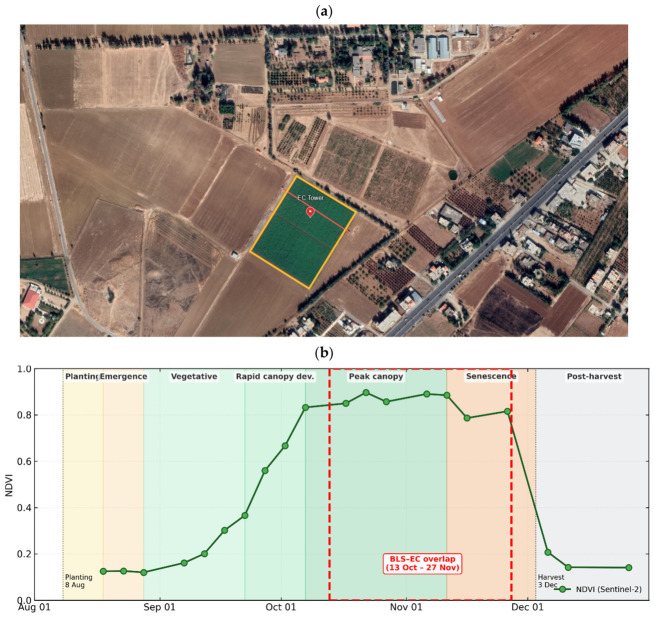
(**a**) Google Earth imagery site map showing field boundaries (yellow), EC location, BLS path orientation (red line), and surrounding land use at the American University of Beirut Agricultural Research and Education Center (AREC), Bekaa Valley, Lebanon (30 October 2021, Google Earth imagery); (**b**) crop phenology from satellite NDVI.

The experiment was conducted on a 1.8 ha potato field at the American University of Beirut’s Agricultural Research and Education Center (AREC) in the Beqaa Valley, Lebanon (33°55′16.3″ N, 36°04′29.0″ E; 995 m a.s.l.) (Figure 1a). The Beqaa Valley has a semi-arid Mediterranean climate with cold winters and dry hot summers. Mean annual precipitation is 520 mm, with an average annual grass reference ET of 1300 mm, 70% of which occurs between April and September [24]. The site is almost flat (negligible slope). The soil of the plot was Calcaric Cambisols (clay soil, bulk density = 1.35 g/cc, field capacity = 40%, permanent wilting point = 22%) [25]. Experiment sensors and field images are found in the Supplementary Information File.

### 2.2. Experimental Period

The field experiment was conducted during the 2021 late growing season. The BLS (Scintec BLS900) was installed on 26 August 2021. The EC system became available for this field only from 12 October 2021 (hence the seven-week deployment offset), and operated continuously through harvest on 3 December 2021. Because the intercomparison requires concurrent, co-located measurements, the analysis is restricted to the matched overlap period: 13 October–27 November 2021. The EC system was powered by solar panels and lithium batteries; the BLS system was connected to the farm’s power supply, which was not a limiting factor for data availability, and no battery outages or significant power-related outages were identified. The BLS–EC overlap corresponds to peak canopy (mean NDVI = 0.86, range 0.79–0.90).

Crop phenology (Figure 1b) was tracked using Landsat and Sentinel-2 surface-reflectance observations converted to the Normalized Difference Vegetation Index (NDVI = (ρ_NIR − ρ_RED)/(ρ_NIR + ρ_RED)) over the field polygon. Twenty cloud-free acquisitions spanning 18 August to 26 December 2021 were used. Figure 1 shows the full NDVI trajectory and resolves the principal phenological stages: emergence (NDVI ≈ 0.12–0.13, 18–28 August), early vegetative growth (NDVI 0.12 → 0.36, 28 August–22 September), rapid canopy development (NDVI 0.36 → 0.85, 22 September–7 October), peak canopy (NDVI ≥ 0.85, 7 October–11 November), senescence onset (NDVI decline after 11 November), and post-harvest bare soil (NDVI ≈ 0.14–0.21 from 6 December onward).

### 2.3. Crop Management

Potato tubers (Agria variety) were planted on 8 August 2021 at 30 cm within-row and 75 cm inter-row spacing, at 25 cm depth. Fertilization at planting comprised 500 kg ha^−1^ of DAP plus 250 kg ha^−1^ of 15–15–15 NPK with trace elements; urea was applied at 300 kg ha^−1^ during hilling (21 September). Irrigation was applied at 7-day intervals in two phases. Phase 1 (sprinkler, 23 August–20 September): five applications (23 August, 30 August, 6 September, 13 September, 20 September) using a sprinkler system with 12 × 18 m spacing, operating at 3.6 bar and a flow rate of 1.83 m^3^ hr^−1^; 9.6 mm applied per event, totaling 48 mm. Phase 2 (micro-sprinkler, 1 October–10 November): six applications (1, 8, 15, 22, 29 October; 5 November) using micro-sprinklers at 130 L hr^−1^ per emitter, 5 × 5 m spacing with lateral overlap, operating at 2.5 bar (main pipe 90 mm, sub-main 75 mm, laterals 32 mm; 15 emitters per lateral); 25 mm applied per event, totaling 150 mm (confirmed by two flow meters and a field rain gauge). Two pressure gauges—one at the valve and one at the terminal lateral—monitored application uniformity throughout Phase 2. The rain gauge recorded 191 mm total water input over Phase 2 and the overlap period (150 mm irrigation + 41 mm rainfall); including Phase 1 sprinkler irrigation (48 mm measured separately), the overall seasonal water input was 239 mm. Potatoes were harvested on 3 December 2021 (31 t ha^−1^ first-grade, 16 t ha^−1^ second-grade).

### 2.4. Instrumentation

Sensors were installed relative to canopy height unless specified. To ensure rigorous comparison and avoid spatial discrepancies in residual calculations, the net radiometer and soil heat flux plates were strictly co-located on the same tripod as the EC system. Table 1 summarizes the instrumentation.

All eddy-covariance instruments were manufactured by Campbell Scientific (Logan, UT, USA), with the exception of the CNR4 net radiometer (Kipp & Zonen, Delft, The Netherlands) and the HFP01 soil heat flux plates (Hukseflux, Delft, The Netherlands). The BLS900 scintillometer and its aperture reducer were manufactured by Scintec AG (Rottenburg, Germany).

Potato canopy height during the intercomparison period ranged from approximately 0.35 to 0.5 m, consistent with peak-canopy development (NDVI ≥ 0.85). Sensor heights above the canopy were maintained approximately constant; adjustments were not required, given the slow canopy growth rate during the late season.

### 2.5. Flux Measurements and Processing

#### 2.5.1. Eddy Covariance

The IRGASON integrated open-path CO_2_/H_2_O gas analyzer and 3D sonic anemometer measured turbulent fluctuations of wind velocity (u, v, w), sonic temperature (T_s), water-vapor density (ρ_v), and CO_2_ concentration at 10 Hz. Standard spectral corrections within the EasyFlux DL processing chain were applied to account for high-frequency attenuation [6]. Fluxes were processed on-site using the Campbell Scientific EasyFlux DL CRBasic program on the CR1000X datalogger (Campbell Scientific, Logan, UT, USA), which applies double coordinate rotation, sonic temperature correction for humidity [26], and the Webb–Pearman–Leuning (WPL) density correction [27]. Quality flags were assigned following Foken et al. [28]; periods with flags indicating poor stationarity or integral turbulence characteristics were excluded. EC fluxes were not closure-corrected during processing; closure is handled separately via the seasonal correction factor CF (Section 3.3), with both uncorrected EC and closure-corrected EC reported throughout as bracketing reference cases. Net radiation (Rn) was measured by a four-component CNR4 net radiometer (Equation (1)). Soil heat flux (G) was calculated from three heat flux plates at 8 cm depth, corrected for heat storage in the overlying layer (Equation (2)).

Net radiation (Rn) was measured by the four-component net radiometer as (Equation (1)):

(1) Rn=(S↓+L↓)−(S↑+L↑)

where S and L are short- and long-wave radiation, respectively.

Soil heat flux (G) was calculated from the average of three heat flux plates at 8 cm depth, corrected for heat storage in the overlying soil layer using co-located TCAV soil temperature and CS655 volumetric water content probes (Campbell Scientific, Logan, UT, USA) (Equation (2)):

(2) Gsurface=Gplate+CsΔtΔTsoilΔz

where Cs is the soil volumetric heat capacity (J m^−3^ K^−1^) estimated from mineral and water fractions, ΔTsoil is the change in mean soil temperature above the plate, and Δz is the plate depth.

#### 2.5.2. Reduced-Aperture Scintillometer (BLS)

The refractive-index structure parameter (Cn^2^) was derived from:

(3) $$C_{n}^{2}=1.12\sigma_{\ln I}^{2}\;D^{\frac{7}{3}}L^{-3}\;\;\;\;\;(m^{-\frac{2}{3}})$$

where σ^2^_ln I_ is the variance of the log-intensity fluctuations, D is the effective aperture diameter (m), and L is the path length (m).

The temperature structure parameter (CT^2^) was obtained following the approach of Andreas [29] and Moene [18]:

(4) $${CT}^{2}\;=\;Cn^{2}\;\times \;(T^{2}/(-0.78\;\times \;10^{-6}\;P){)}^{2}\;\times \;(1\;+\;0.03/\beta {)}^{-2}$$

where T is air temperature (K), P is atmospheric pressure (Pa), and β is the Bowen ratio. The Bowen ratio was estimated iteratively from preliminary estimates of H and LE derived from the energy balance [17]. The humidity-correction term (1 + 0.03/β)^−2^ accounts for refractive-index fluctuations caused by humidity [18]; this correction is small when β is not close to zero.

Sensible heat flux was derived iteratively using MOST:

(5) $$C_{T}^{2}=C_{n}^{2}\;{\left(\frac{T^{2}}{-0.78*10^{-6}}*200\right)}^{2}{\left(1+\frac{0.003}{\beta }\right)}^{-2}$$

(6) $$\frac{C_{T}^{2}}{T_{*}^{2}}{\left(Z_{LAS}-d\right)}^{\frac{2}{3}}=f_{T}(\frac{Z_{LAS}-d}{L})\;$$

(7) $$T*=-\frac{H}{\rho \;C_{p}u*}$$

(8) $$u*=\frac{ku}{\ln\left[\frac{z_{u}-d}{Z_{0m}}\right]-\varphi_{m}\left[\frac{\left(z_{u}-d\right)}{L}\right]+\varphi_{m}(\frac{z_{0m}}{L})\;}$$

where $C_{T}$ is the temperature structure parameter (${K^{2}/m}^{-\frac{2}{3}}$); T is the air temperature (K), $T_{*}$ is the temperature scale (k); P is the air pressure (Pa); β is the Bowen ratio (the ratio of sensible heat flux to latent heat flux); $Z_{RAS}$ is the effective RAS measurement height (m); d is the zero-plane displacement height (m); L is the Obukhov length; $z_{u}$ is the wind measurement height (m); $Z_{0m}$ is the dynamic coarse roughness (m); $f_{T}$ is universal function related to atmospheric stability; H is the sensible heat flux (W m^−2^); ρ is the air density (kg m^−3^); $C_{p}$ is the constant pressure of the specific heat (J kg^−1^ K^−1^); u* is the friction wind speed in meters per second and $\varphi_{m}$ is a modified function of momentum stability, with its integrated form following the parameterization of Businger et al. [30].

The universal temperature function f_T_ was parameterized according to De Bruin et al. [31]. The coupled system of Equations (5)–(8) was solved iteratively for each 30 min period. Starting from a neutral first guess (L → ∞), the scheme alternately updated the temperature scale T* (Equation (7)) and friction velocity u* (Equation (8)) and recomputed the Obukhov length L = u*^2^T^−^/(k g T*); iteration continued until the change in Obukhov length between successive steps satisfied |L_Ob − L_Ob, prev| < 0.1 m, following the convergence criterion documented in the Scintec Theory Manual [32] and the standard iterative MOST flux–profile scheme [30,31]. Convergence typically required 3–4 iterations under daytime unstable conditions and up to 8–10 iterations under near-neutral conditions; non-converging periods (<1.5% of daytime records) were discarded. The humidity-correction term (1 + 0.03/β)^−2^ is sensitive to small β: with more than half of daytime periods having β < 0 and ~10% with |β| < 0.1, this introduces a structural ~30% uncertainty in H_BLS whenever β is near zero [18] (see Section 4.2).

Wind speed, wind direction, air temperature, and temperature gradient were measured at the BLS receiver station. For the residual-energy balance calculation, latent heat from the BLS (LE_BLS) was obtained using the same Rn and G measured at the EC tower (Equation (9)), ensuring consistency in available energy across methods:

LE_BLS = Rn − G − H_BLS(9)

#### 2.5.3. Gap-Filling of BLS Sensible Heat Flux

Over the 46-day BLS–EC overlap period, 4290 fifteen-minute BLS records were available, of which 1647 (38.4%) yielded valid H estimates; the remaining 2643 periods were gap-filled using Marginal Distribution Sampling (MDS) [33], which stratifies by Rn, air temperature, and wind speed within a moving time window. A secondary Mean Diurnal Variation (MDV) fill was also computed for uncertainty assessment. The two methods agree with RMSE = 14.3 W m^−2^ for the gap-filled periods in the overlap window. The gap rate was strongly diurnal—exceeding 90% at night but remaining below 15% during midday—so the impact on daytime ET totals is limited. All ET statistics use daytime data only (Rn > 0).

### 2.6. Flux Footprint Analysis

The flux footprint of the EC system was estimated using the analytical model of Kljun et al. [34]. The model was applied to each 30 min period using measured friction velocity (u*), standard deviation of lateral wind velocity (σ_v), mean wind speed, and Obukhov length from the EC sonic anemometer, with displacement height and roughness length estimated from canopy height (d = 0.67 h_c; z_0 m = 0.1 h_c). Boundary-layer height was estimated as h = 0.25 u*/f, where f is the Coriolis parameter at 33.9° N. The EC tower was located 56 m from the nearest field edge; the BLS path extended 140 m along an azimuth of 119°.

The BLS measures the path-averaged structure parameter of temperature (C_T^2^) along the optical beam. Each point’s contribution is weighted by the bell-shaped path-weighting function:

(10) $$W(u)=16.7\cdot u^{5/3}(1-u{)}^{5/3}$$

where u = x/L (normalized position along the path, 0 to 1), peaking at the path center. The 2-D BLS footprint was computed by weighting the crosswind-integrated footprint at each path point by W(u) and integrating along the path:

(11) $$f_{LAS}(x,y)=\int_{0}^{L}W(u)\cdot f_{point}(x,y|\mathrm{position}\;u)\;du$$

### 2.7. Sensible Heat Flux Intercomparison

H from the two methods was compared at 30 min, hourly, daily, and weekly scales over the overlap period, with quality-control criteria applied separately for each instrument.

For the EC system, half-hourly fluxes were retained at EddyPro quality flags 0–8 [28]; (stationarity and integral turbulence tests), discarding only the flag-9 reject class. A hard threshold of u* > 0.02 m s^−1^ was additionally imposed to exclude instrument noise-floor periods; this removed only 11 of 1298 daytime half-hourly periods (0.3%). No further u* screening was applied to the daytime record, consistent with the established position that fixed u* filtering targets nighttime carbon-flux underestimation [35,36] rather than daytime sensible- and latent-heat-flux QC, where flux quality is governed by the Foken et al. [28] flag system.

For the BLS, only records with SRun error codes 0 or 8192 (valid data) were retained. SRun’s internal signal-quality and log-normality checks reject periods of weak turbulence, which—together with routine nighttime stable conditions—account for the 61.6% overall BLS gap fraction; the nighttime contribution dominates (gap fraction exceeding 90% at night), so the daytime data loss relevant to the intercomparison is substantially smaller than the overall figure implies.

For both methods, a physical-plausibility filter |H| < 500 W m^−2^ was applied, and daily and weekly aggregates required ≥10 valid 30 min values per day.

The daytime EC record included a non-trivial low-turbulence fraction: 20.8% of daytime half-hours had u* < 0.1 m s^−1,^ and 52.0% had u* < 0.2 m s^−1^. Removing these periods did not materially change the daytime EC statistics. Weak turbulence is also a known source of bias in scintillometer-derived C_n_^2^: the Scintec Theory Manual [32], reports that for the D = 0.15 m aperture, C_n_^2^ overestimation grows from 5% to 20% as the inner scale of turbulence l_0_ increases from 0.01 to 0.02 m (l_0_ = 0.01 m corresponding to u* ≈ 0.05 m s^−1^ at 2 m height), and Van Kesteren et al. [37] report up to 30% C_n_^2^ overestimation at u* < 0.2 m s^−1^ owing to neglect of the dissipation range of the refractive-index spectrum. In the absence of a path-integrated turbulence measure, we use the EC tower u* as a proxy for turbulence over the BLS path: only 5.2% of daytime periods fell below the manual’s critical u* ≈ 0.05 m s^−1^ threshold, and most of these were already removed by SRun’s internal quality filter. The residual influence of low-turbulence C_n_^2^ overestimation on the daytime BLS H comparison is therefore expected to be small.

To make the basis of each comparison explicit, the matched analyses draw on the following sample sizes. The BLS–EC overlap spans 46 calendar days (13 October–27 November 2021). Daily and cumulative ET statistics are reported for the 45 days on which at least ten valid daytime (Rn > 0) 30 min periods were available for both methods; one day failing this criterion was excluded. The half-hourly sensible-heat-flux intercomparison uses *n* = 596 daytime periods in which both instruments returned valid, non-gap-filled H, aggregating to *n* = 332 hourly and *n* = 42 daily values. The daily H sample is smaller than the daily ET sample because it additionally requires simultaneously valid, non-gap-filled retrievals from both instruments within each averaging period, whereas the residual ET comparison admits MDS-gap-filled BLS H. The energy-balance-closure regression and the daily mean H time series are instead evaluated over all 46 overlap days, because a daily mean of the closure terms and of H can be formed whenever a day contains valid periods; these 46-day daily means therefore admit MDS-gap-filled BLS H, unlike the strictly non-gap-filled 42-day daily H. The Bowen-ratio distribution (Section 3.3) is based on 2108 quality-controlled 30 min periods, and the half-hourly LE comparison on 435 daytime hours. Because several of the daily scale comparisons rest on only tens of independent days, the corresponding regression slopes, correlation coefficients, and mean bias errors carry appreciable sampling uncertainty and are interpreted as indicative of systematic behavior rather than as precise population estimates; this small-sample limitation is revisited in Section 5.

### 2.8. Statistical Analysis

Agreement was evaluated using: Nash–Sutcliffe efficiency (NSE, Equation (12)); percentage bias (PBIAS, Equation (13)); Pearson correlation coefficient r (Equation (14)); coefficient of determination R^2^ = r^2^ (i.e., the square of r, Equation (15), corrected from earlier erroneous typesetting); root mean square error (RMSE, Equation (16)); and mean bias error (MBE, Equation (17)). For ET comparisons, the Bowen-ratio-preserving correction [22] and the residual-to-LE [23] correction are both reported as separate EC reference cases.

(12) $$NSE=1-\frac{{\sum_{i=1}^{n}\left(a_{i}-p_{i}\right)}^{2}}{{\sum_{i=1}^{n}\left(a_{i}-a_{avg}\;\right)}^{2}}$$

(13) $$PBIAS=\frac{\sum_{i=1}^{n}\left(p_{i}-a_{i}\right)}{\sum_{i=1}^{n}a_{i}}$$

(14) $$r=\sum_{i=1}^{n}\frac{\left(a_{i}-a_{avg})(p_{i}-p_{avg}\right)}{{\left(a_{i}-a_{avg}\right)}^{2}{\left(p_{i}-p_{avg}\right)}^{2}}$$

(15) $$R^{2}=r^{2}$$

(16) $$RMSE=\sqrt{\frac{1}{n}(\sum_{i=1}^{n}{\left(a_{i}-p_{i}\right)}^{2})}$$

(17) $$MBE=\frac{1}{n}\sum_{i=1}^{n}\left(p_{i}-a_{i}\right)$$

where $a_{i}$ is the observed (EC) value, $p_{i}$ is the predicted (BLS) value, *a_avg_* and *p_avg_* are the respective means, and *n* is the number of observations.

## 3. Results

### 3.1. Meteorological Conditions

**Figure 2:**
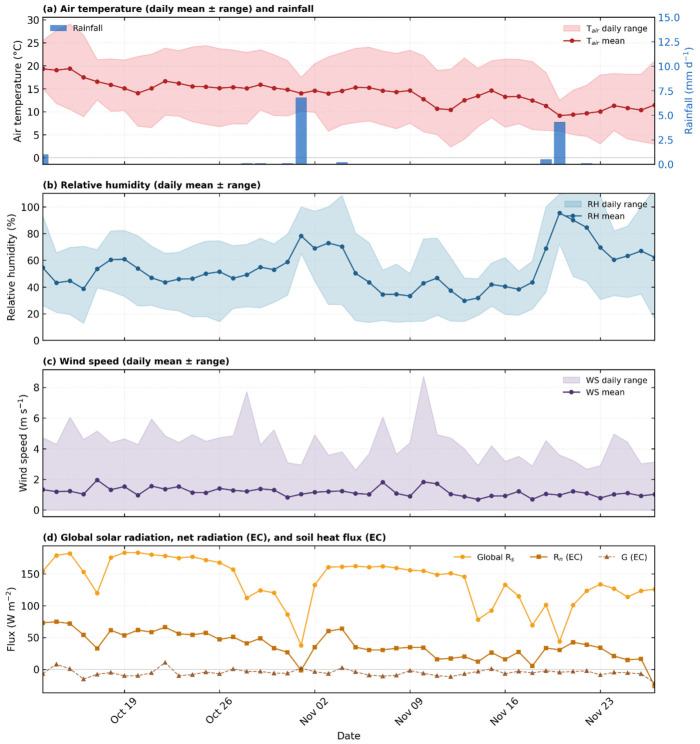
Meteorological conditions during the BLS–EC overlap period (13 October–27 November 2021): (**a**) air temperature (BLS and EC); (**b**) relative humidity (BLS and EC); (**c**) wind speed (BLS and EC); (**d**) net radiation and soil heat flux (EC). EC-measured Rn is used throughout this study for the available energy calculation.

Meteorological conditions during the intercomparison period reflect a late-autumn transition in the Beqaa Valley (Figure 2). Data are taken from the AREC on-site weather station (CR1000 15 min records aggregated to daily statistics: mean, minimum and maximum). Daily mean air temperature declined from ~19 °C in mid-October to ~10 °C in late November, with daily minima reaching as low as 2.3 °C during a cold spell in late October and maxima as high as 29 °C on the warmest autumn day. Mean relative humidity was 53% (daily minima 13%, daily maxima near 100% on rainy mornings) with a clear drying tendency in the central part of the overlap. The IRGASON and sonic anemometer operate reliably below 0 °C, and the coldest pre-sunrise periods are independently excluded by both the EC quality-flag screen and the BLS signal-strength filter, so cold-morning artifacts are not present in the daytime record. The overlap period recorded 13.2 mm of rainfall distributed over five rainy days, with the largest event of 6.8 mm on 1 November; this falls between the end of micro-sprinkler irrigation (10 November) and the harvest (3 December). Daily mean global solar radiation averaged 138 W m^−2^, declining from ~180 W m^−2^ in mid-October to ~110 W m^−2^ by late November as solar elevation and day length decreased. EC-measured daily mean net radiation followed the same pattern, declining from ~75 W m^−2^ to ~20 W m^−2^. Daily mean wind speed was 1.2 m s^−1^, with daily maxima reaching 4–9 m s^−1^ on frontal passages. Mean daytime albedo was 0.20 ± 0.05, consistent with a dense, fully developed canopy (mean NDVI = 0.85; Figure 1b) that was beginning to senesce in the last 10 days of the record.

### 3.2. Wind Regime and Atmospheric Stability

**Figure 3:**
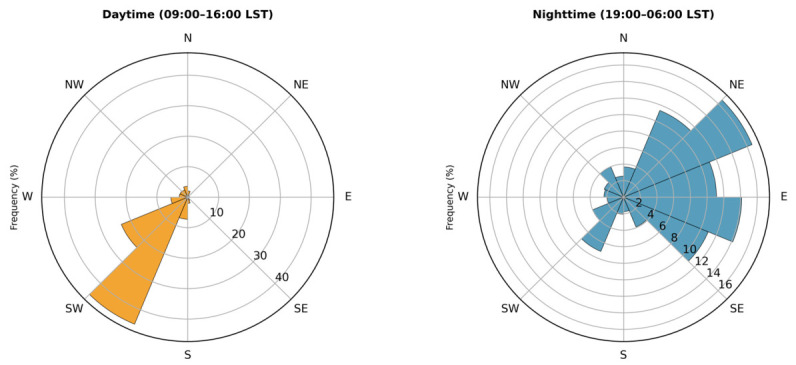
Wind-direction distribution at the BLS site. (**Left**): core daytime hours (09:00–16:00 LST), chosen to isolate the established up-valley regime, dominated by SSW flow (45.1% from SSW, 23.3% from SW). (**Right**): nighttime (19:00–06:00 LST), dominated by NE drainage flow (16.6% NE, 14.1% E, 11.4% NNE, 11.1% ENE). Note: flux statistics in the text use a separate daytime definition (Rn > 0, ≈07:00–17:00 LST).

The wind-direction record reveals the characteristic bimodal regime of the Beqaa Valley (Figure 3). Daytime flow was SSW-dominated (circular mean 227°, computed using circular statistics [38]), delivering up-valley air masses to the site, while nighttime flow reversed to a weaker, more distributed NE–ESE drainage flow (circular mean 79°; the four leading sectors together account for 53% of nighttime observations). The prevailing daytime direction is favorable for both instruments: fetch in the SSW sector exceeds 110 m, giving a fetch-to-height ratio of ≈50:1 at the BLS effective height (z_eff = 2.15 m; Section 2.6), adequate for flux measurement over this surface. The wind also crossed the BLS path (azimuth 119°) near-perpendicularly—227° flow against a 119° path axis gives an incidence of ≈ 108°—so the crosswind component dominated during the hours of largest H, the favorable geometry for path-averaged scintillometry.

Atmospheric stability over the overlap period was approximately evenly divided between unstable and stable regimes: 60% of daytime periods were unstable (L < 0; median L = −27 m) and 40% stable (L > 0; median L = +40 m). A substantial fraction lay near neutral—31% of daytime periods had |L| > 100 m (|ζ| <≈ 0.02 at z_eff = 2.15 m)—so the high stable count reflects predominantly weakly stable, near-neutral conditions rather than strongly stratified flow. The BLS-derived friction velocity had a median of 0.215 m s^−1^ (IQR 0.131–0.341 m s^−1^), with a clear diurnal cycle peaking near 0.35 m s^−1^ in the early afternoon.

### 3.3. Energy-Balance Closure

**Figure 4:**
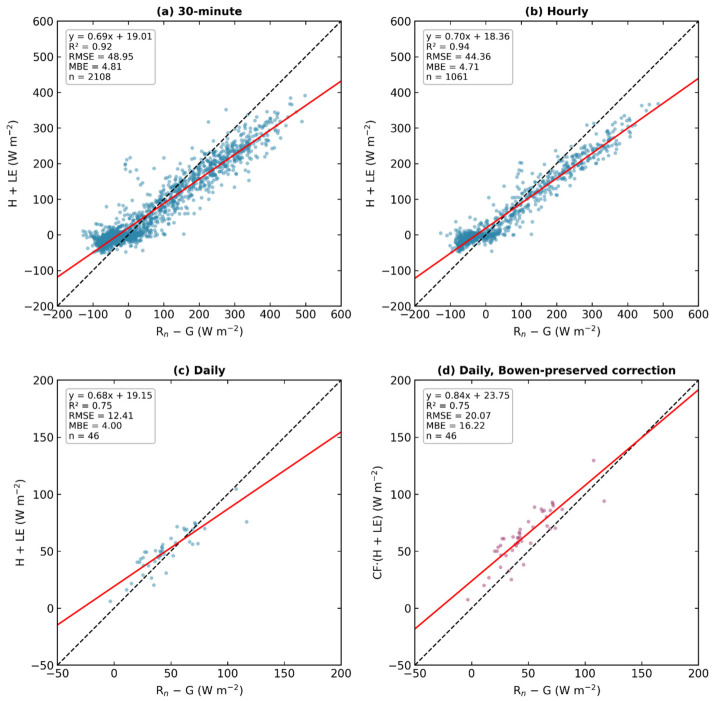
Energy-balance closure at (**a**) 30 min, (**b**) hourly, (**c**) daily, and (**d**) daily with seasonal Bowen-ratio-preserved correction. Dashed line: 1:1; solid red: OLS regression. The closure deficit (≈19%) is taken up almost entirely by the correction.

Energy-balance closure was evaluated by comparing available energy (Rn − G) with turbulent fluxes (H + LE) from the EC system at multiple temporal scales (Figure 4). The closure regression slope was stable across temporal scales—0.69 at the 30 min scale (R^2^ = 0.92), 0.70 hourly (R^2^ = 0.94), and 0.68 daily (R^2^ = 0.75; the lower R^2^ at the daily scale reflects the small number of days, *n* = 46)—while the systematic deficit persisted. The seasonal daytime Bowen-ratio-preserved correction factor [22] is computed from the aggregated daytime flux sums over the full BLS–EC overlap period (Rn > 0) as CF = Σ(Rn − G)/Σ(H + LE) = 1.24; this single seasonal value is the one applied in all closure-corrected analyses reported below. It is obtained from summed (energy-weighted) fluxes rather than from the arithmetic mean of monthly factors, and the corresponding ~19% closure deficit falls within the range commonly reported for agricultural surfaces [6,10]. For transparency, the same sum-based ratio computed within each month gives CF = 1.26 in October (*n* = 335 daytime 30 min periods) and CF = 1.22 in November (*n* = 474 periods). October and November contributed nearly equal shares of daytime available energy (51% and 49%, respectively), so the energy-weighted seasonal value (1.24) coincides almost exactly with the simple two-month mean (1.24); the aggregated sum nonetheless remains the physically appropriate basis for correcting cumulative ET, since it would weight months correctly were their energy contributions unequal. Relative to the seasonal factor, the monthly values imply a slight under-correction in October (true CF = 1.26 > 1.24) and a slight over-correction in November (true CF = 1.22 < 1.24); the sensitivity of ET estimates to this monthly variation is examined in Section 4.2.

**Figure 5:**
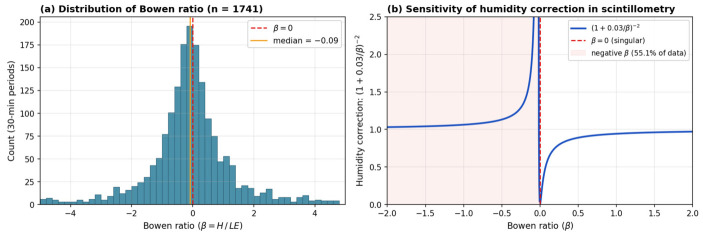
(**a**) Distribution of Bowen ratio (β = H/LE) for valid 30 min periods during the BLS–EC overlap; the distribution is centered near zero (median −0.11). (**b**) Sensitivity of the humidity-correction term (1 + 0.03/β)^−2^ in the BLS C_T^2^–C_n_^2^ conversion; the term diverges as β → 0.

A defining characteristic of this late-season, well-irrigated dataset is the predominance of negative and near-zero Bowen ratios: 54.7% of 30 min periods had β < 0 and 10% had |β| < 0.1 (Figure 5). Under these conditions, the humidity-correction term (1 + 0.03/β)^−2^ applied in the C_T^2^–C_n_^2^ conversion (Equation (4)) becomes highly sensitive and diverges as β approaches zero. Although the iterative Bowen-ratio estimation in the Scintec SRun software (v. 1.0) typically stabilizes, uncertainty in β propagates non-linearly into H_BLS whenever β is small. The Bowen-ratio-preserving closure correction should therefore be applied with caution: when β oscillates around zero, multiplicative scaling can redistribute residual energy unpredictably between H and LE. For this reason, we restrict the closure correction to periods with Rn > 0 and report results against both uncorrected and closure-corrected EC as bracketing reference cases.

### 3.4. Footprint Representativeness

**Figure 6:**
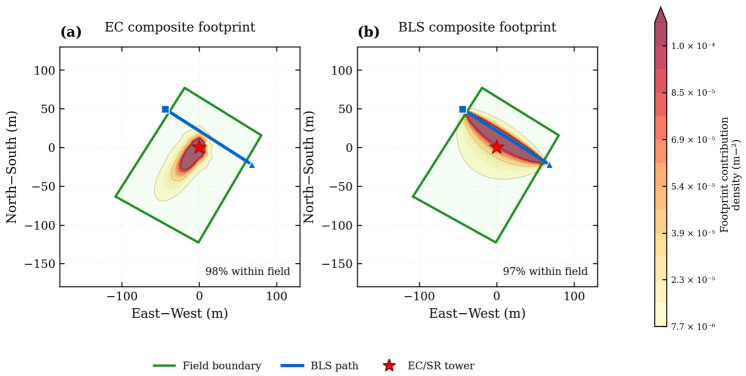
Flux footprints for (**a**) the eddy-covariance (EC) tower and (**b**) the reduced-aperture scintillometer (BLS) over the potato field.

Footprint analysis confirmed that both EC and BLS measurements predominantly sampled the target potato field (Figure 6). EC footprints were computed with the Kljun et al. [34] two-dimensional parameterization (inputs: measurement height z_m, roughness length z0, u*, σ_v, and boundary-layer height), and the BLS path-footprint was obtained by convolving the path-weighting function with a point footprint following [39]. Under prevailing SSW winds, the EC footprint was concentrated in the central and southwestern portions of the field: the 90% cumulative contribution fell within the field boundary for 91.3% of individual daytime periods and for 98% of the probability-weighted composite. The peak (modal source distance) of the EC footprint lay ≈ 14 m upwind of the tower, and the 80% cumulative source area, though extending farther downwind, remained within the field; this modal distance is about one-quarter of the minimum tower-to-edge distance (56 m).

The BLS footprint extended downwind along the beam path, reflecting its path-integrated nature, yet still retained 85.8% of cumulative contribution within the field for individual periods (97% for the composite). The ≈14% out-of-field contribution under SSW flow originated from surface downwind of the path—bare soil; because this surface contrasted with the irrigated canopy, it represents a minor and negligible source of bias.

### 3.5. BLS vs. EC Sensible Heat Flux

**Figure 7:**
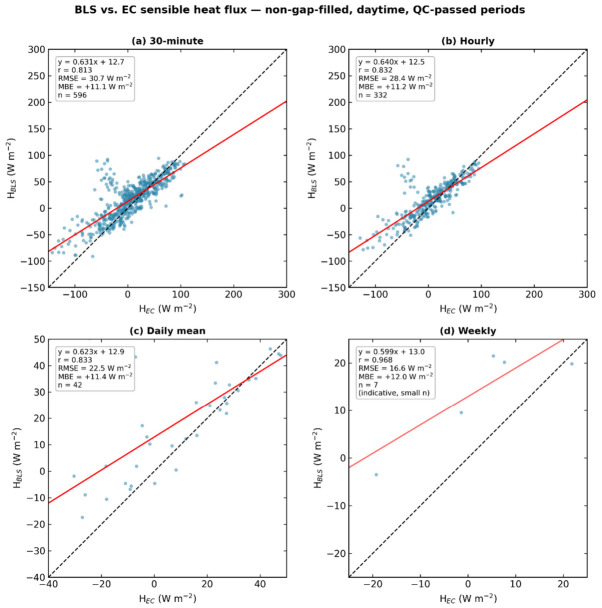
BLS vs. EC sensible heat flux at four temporal scales during the overlap period (13 October–27 November 2021): (**a**) 30 min (n = 596 matched valid pairs), (**b**) hourly (*n* = 332), (**c**) daily (*n* = 42), (**d**) weekly (*n* = 7, indicative). Sample sizes are matched daytime periods in which both instruments returned valid, non-gap-filled retrievals (non-gap-filled BLS H -error codes 0/8192, both 15 min samples valid) with simultaneous QC-passed EC H, daytime Rn > 0—(Section 2.7). Dashed line: 1:1; solid red: OLS regression.

BLS H was evaluated against EC across four temporal scales (Figure 7). The 30 min comparison uses *n* = 596 matched daytime periods in which both instruments returned valid, non-gap-filled H passing Section 2.7 quality control. This is far fewer than the calendar daytime slots over the 46-day window because (i) the BLS retrieval is valid in only 74.3% of daytime periods, and gap-filled values are excluded from the intercomparison, and (ii) a simultaneously valid EC retrieval, requiring both constituent 15 min sub-periods, is additionally required. The 596 pairs are a subset of the 2108 quality-controlled periods used for the Bowen-ratio distribution (Section 3.3), which do not require a simultaneously valid paired retrieval.

Across scales, correlation was stable (r~0.82) while RMSE fell with aggregation (30.7 → 28.4 → 22.5 → 16.6 W m^−2^) and the regression slope remained near-constant (0.631 at 30 min, 0.640 hourly, 0.623 daily, 0.599 weekly). This scale-invariant slope below unity, combined with high correlation, is the signature of a systematic amplitude offset rather than random error: aggregation averages out scatter but cannot remove a proportional bias. At the weekly scale (*n* = 7), the correlation is high (r = 0.968) but rests on only seven aggregated observations and should be regarded as indicative rather than robust, since small samples can yield inflated and unstable r values; it is reported only to illustrate the effect of temporal aggregation on random scatter. The sub-unity slope means the BLS underestimates H at the high-flux end relative to EC, whereas the daily mean bias is positive (MBE = +11.4 W m^−2^); these are reconciled in the next paragraph and are not contradictory.

This amplitude offset is not a free-convection approximation artifact. The Scintec Theory Manual [32] states that the free-convection approximation is valid only for ζ < −0.2 and underestimates H by more than 10% beyond this threshold; our processing instead uses the full MOST framework (Equations (5)–(8)) with measured wind speed. The median ζ over unstable daytime periods, ζ = z_eff/L = 2.15/(−27) ≈ −0.08, lies well inside the MOST-valid regime (ζ > −0.2), confirming that the persistent ≈ 0.63 slope is not a stability-framework artifact. The sensor-to-canopy ratio likewise satisfies the manual’s 2–5× roughness-element criterion (Section 2.2): z_sensor/h_c = 2/0.5 = 4.

**Figure 8:**
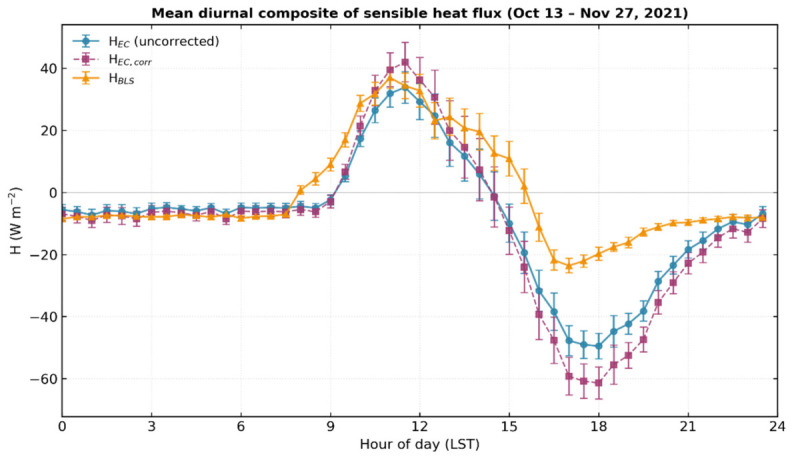
Mean diurnal composite of sensible heat flux from EC (blue), EC with seasonal Bowen-preserving correction (CF = 1.24, purple dashed), and BLS (orange). Error bars: standard error of the mean.

**Figure 9:**
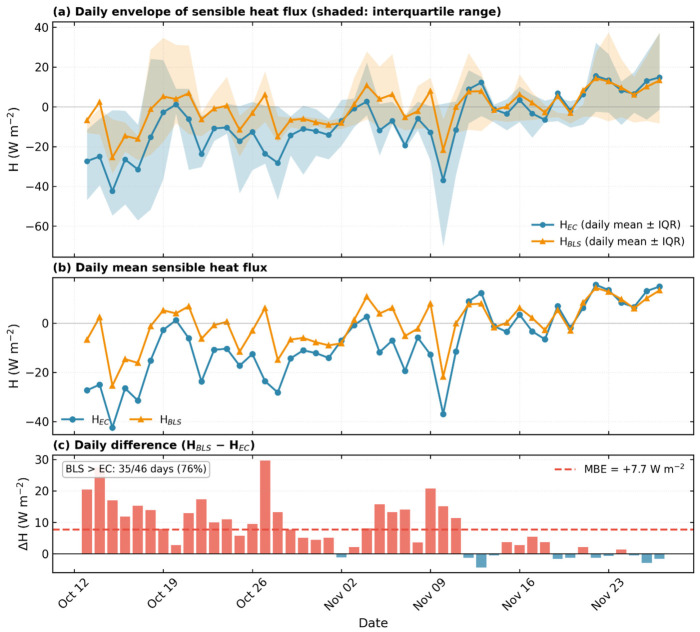
Time series of sensible heat flux (H) from eddy covariance (EC) and the reduced-aperture scintillometer (BLS) during the overlap period (13 October–27 November 2021). (**a**) Daily mean H with interquartile range (IQR, shaded bands) showing the within-day variability of 30 min values. (**b**) Daily mean H for both methods. (**c**) Daily difference (H_BLS − H_EC). Red bars indicate days when BLS exceeded EC; blue bars indicate the reverse. The dashed line marks the daily mean bias error.

The direction of the bias is regime dependent. At the midday H-peak (09:00–14:00 LST), the EC, Bowen-corrected EC, and BLS curves agree closely (Figure 8); the positive daily MBE is instead carried by the low- and negative-H regime, where the BLS does not track EC’s strong negative excursions. During the evening transition (16:00–20:00 LST), as daytime convection decays into nighttime stability, uncorrected EC reaches ≈−55 W m^−2^ while the BLS descends only to ≈−25 W m^−2^ (Figure 8). The same asymmetry appears in the time series (Figure 9): the BLS envelope is narrower and positively offset, BLS exceeds EC on 76% of days, and the offset is largest in mid-October when daily mean H is most negative, shrinking toward late November as H approaches zero in advanced senescence. Thus, the sub-unity slope (BLS lower at high H) and the positive MBE (BLS higher where EC goes strongly negative) are two facets of the same behavior: relative to EC, the BLS compresses the H range. This compression is consistent with path-averaging and differences in turbulent sampling between the two instruments, which act to reduce the high-flux extremes recorded by the BLS relative to the point EC sensor. The EC energy-balance-closure deficit (≈19%), by contrast, depresses EC turbulent fluxes overall and therefore contributes to the positive mean offset rather than to the amplitude compression [40,41].

Figure 8 shows the mean diurnal composite of sensible heat flux from EC, EC with seasonal Bowen-preserving correction, and BLS. The three curves agree closely during the midday H-peak (09:00–14:00 LST). The most visible divergence occurs during the evening transition (16:00–20:00 LST), when uncorrected EC exhibits a strong negative excursion (down to −55 W m^−2^) that the BLS only partially reproduces (down to −25 W m^−2^). This difference during the stable-to-unstable transition likely reflects rapid stability changes affecting the sonic anemometer’s turbulence statistics and advective heat influx as the wind direction reverses.

Figure 9 shows the time series of sensible heat flux at 30 min (range) and daily mean resolution during the overlap period. The EC envelope extends further into negative values, reflecting strong nighttime cooling fluxes over the irrigated surface, while the BLS envelope is narrower and positively offset (Figure 9a). BLS consistently exceeds EC, with the offset largest in mid-October (when daily mean H is most negative) and smallest in late November as H approaches zero during advanced senescence (Figure 9b). The persistent positive bias is consistent with the EC energy-balance closure deficit (~19%) and diminishes as sensible heat flux magnitudes decrease toward the end of the study period (Figure 9c).

### 3.6. Latent Heat Flux and Evapotranspiration

**Figure 10:**
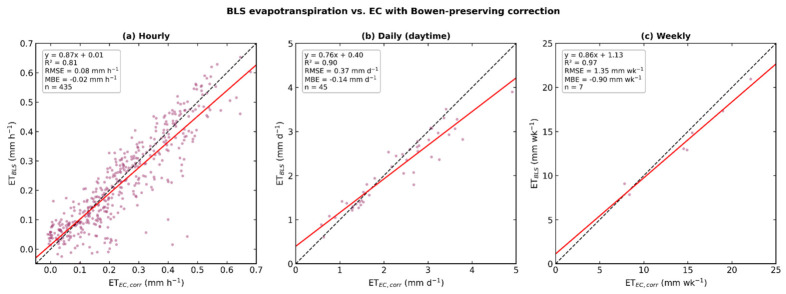
Comparison of BLS (residual-method) evapotranspiration against closure-corrected EC (Bowen-ratio-preserving correction, CF = 1.24) at three temporal scales: (**a**) hourly (*n* = 435), (**b**) daily (*n* = 45), (**c**) weekly (*n* = 7). Dashed line: 1:1; solid red: OLS regression.

BLS-derived latent heat flux was computed as the residual of the surface energy balance using EC tower Rn and G. Comparison with EC LE over 435 daytime hours yielded a strong correlation (r = 0.90) but a systematic positive bias against uncorrected EC attributable to the closure gap (Table 2). After the seasonal CF = 1.24 was applied to EC LE, the systematic bias was substantially reduced, with close agreement at the daily scale (MBE = −0.14 mm d^−1^; Figure 10, Table 2). Because both BLS residual LE and closure-corrected EC LE share the same Rn and G, their agreement reflects internal consistency rather than fully independent validation and is stated as such throughout.

Table 2 compares BLS ET against EC ET under three closure assumptions for daytime periods. The Mauder correction assigns the full energy-balance residual to LE (H unchanged), while the Twine correction scales both H and LE proportionally. Because Mauder-corrected EC LE and BLS LE are both computed as residuals from the same Rn and G, the superior Mauder–BLS agreement is partly non-independent; the Twine correction is therefore the preferred reference for this dataset.

Over the matched period, cumulative ET was 82.5 mm (EC uncorrected), 102.3 mm (EC Twine-corrected), and 96.0 mm (BLS) (Table 2). BLS exceeds uncorrected EC by +16% but is 6% below closure-corrected EC, indicating that the apparent BLS overestimation relative to uncorrected EC is largely attributable to the EC energy-balance closure deficit.

**Figure 11:**
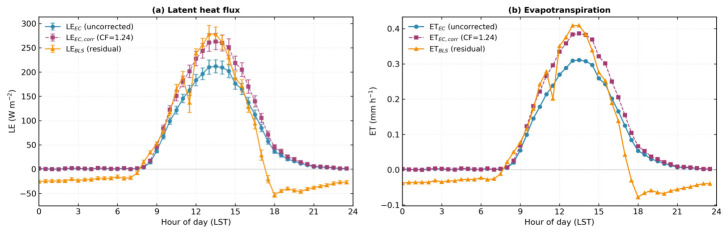
Mean diurnal composite of (**a**) latent heat flux and (**b**) evapotranspiration from the three methods. Closure-corrected EC (purple dashed) and BLS residual (orange).

Figure 11 shows the mean diurnal composite of latent heat flux and evapotranspiration. Closure-corrected EC and BLS residual LE track each other closely throughout the day. Uncorrected EC LE is systematically lower at the midday peak, consistent with the closure deficit. At night, BLS residual LE is slightly negative because, when available energy (Rn − G) is small and frequently changes sign, the residual LE = Rn − G − H_BLS is dominated by the uncertainty in H_BLS and by the small, often counter-gradient stable-stratification fluxes; under these conditions the closure assumption that the residual equals the true latent heat flux does not hold, so the small negative values are a physical and methodological artifact of the residual method at low available energy rather than evidence of downward water-vapor transport. For this reason, daytime-only ET (Rn > 0) is used in all cumulative statistics.

**Figure 12:**
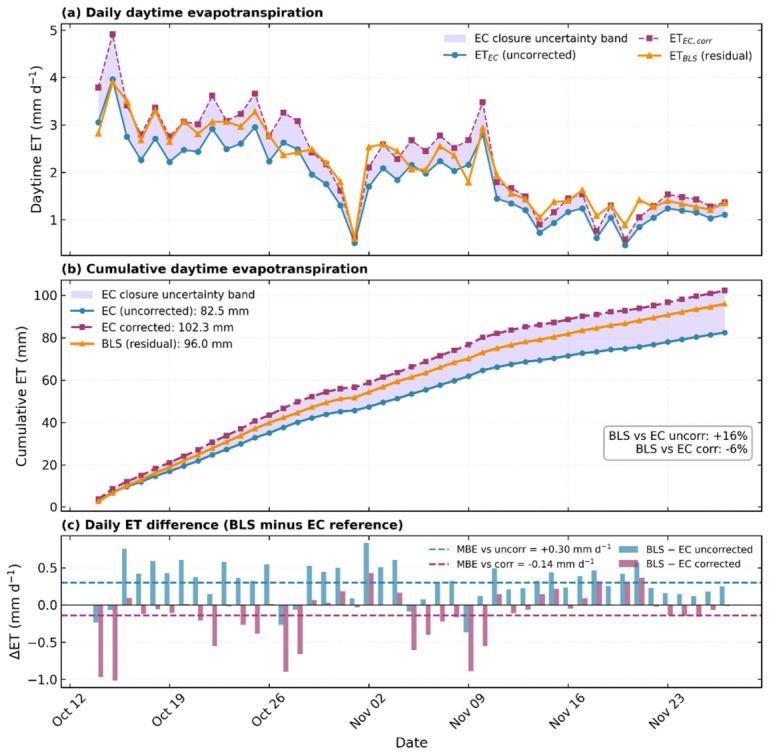
Time series of daytime evapotranspiration from eddy covariance (EC) and the reduced-aperture scintillometer (BLS) over the overlap period (13 October–27 November 2021). (**a**) Daily daytime ET (Rn > 0) from uncorrected EC (blue), closure-corrected EC (Bowen-ratio-preserving correction, CF = 1.24; purple), and the BLS residual method (orange); the shaded band between the two EC curves denotes the energy-balance closure uncertainty, within which the BLS estimate falls on most days. (**b**) Cumulative daytime ET over the matched days: 82.5 mm (uncorrected EC), 102.3 mm (closure-corrected EC), and 96.0 mm (BLS); (**c**) daily ET difference between BLS and each EC reference—BLS minus uncorrected EC (blue) and BLS minus closure-corrected EC (purple); dashed lines mark the corresponding mean bias errors (+0.30 and −0.14 mm d^−1^).

Both systems tracked the seasonal ET decline consistently (Figure 12a), from ~3–4 mm d^−1^ in mid-October to ~1 mm d^−1^ by late November. Over the 45 matched days, cumulative daytime ET was 82.5 mm (uncorrected EC), 102.3 mm (closure-corrected EC), and 96.0 mm (BLS)—BLS exceeding uncorrected EC by 16% but lying 6% below closure-corrected EC (Figure 12b). Daily differences confirm this pattern: MBE = +0.30 mm d^−1^ against uncorrected EC versus −0.14 mm d^−1^ against closure-corrected EC (Figure 12c). The near-zero bias against closure-corrected EC indicates that the apparent BLS overestimation relative to uncorrected EC is largely attributable to the EC energy-balance closure deficit rather than to a scintillometer measurement error.

## 4. Discussion

### 4.1. Energy-Balance Closure and Its Implications

The daily energy-balance closure of 81% falls within the range commonly reported for agricultural EC systems [10,11,12] and is consistent with values obtained over irrigated and rain-fed croplands by Ezzahar et al. [17], Liu et al. [42], and Bambach et al. [6]. The incomplete closure reflects several well-documented factors: limited spatial representativeness of point-scale soil sensors (three heat flux plates at the field center), unaccounted heat storage in the canopy–air layer [43], spectral losses at low frequencies in the EC processing chain [6], and under sampling of mesoscale eddies contributing to flux transport [11,12]. The manufacturer of the scintillometer independently acknowledges this phenomenon: the Scintec Theory Manual [32] notes that the simplified surface energy balance ‘often does not close—instead it shows a gap of approximately 20%’ attributable to storage effects, measurement uncertainties, secondary circulations, and photosynthesis.

The closure residual has fundamental implications for the intercomparison. Because BLS derives LE as a residual of the surface energy balance, it inherently forces energy-balance closure at each timestep and therefore does not share the same underestimation bias as uncorrected EC. Comparing BLS against uncorrected EC thus conflates method-specific biases with the known closure deficit of EC. To bracket the true method comparison, we present results against both uncorrected and closure-corrected EC as reference cases (Table 2).

### 4.2. Closure Correction Methods: Twine vs. Mauder

We compared two established closure correction approaches: the Bowen-ratio-preserving method of Twine et al. [22], which scales both H and LE by CF = Σ(Rn − G)/Σ(H + LE); and the residual-to-LE method of Mauder et al. [23], which assigns the full energy-balance residual to LE while leaving H unchanged. For this dataset, both corrections substantially reduce the BLS–EC ET bias compared to uncorrected EC (Table 2). The Mauder correction yields a smaller RMSE (0.28 vs. 0.35 mm d^−1^) and higher correlation (r = 0.97 vs. 0.95), but this apparent superiority is largely an artifact of non-independence: both Mauder-corrected EC LE and BLS LE are computed as residuals from the same Rn and G, so their agreement reflects shared methodology rather than independent confirmation.

The Bowen-ratio-preserving method [22] is preferred for two reasons. First, it preserves the measured H/LE partitioning rather than channeling the entire closure residual into LE, which is physically unjustified on this well-irrigated surface where both turbulent fluxes carry substantial fractions of available energy. Second, it retains the independently measured H in the reference, avoiding the circularity that inflates the Mauder–BLS agreement.

The correction must also be applied with caution in datasets where the Bowen ratio oscillates around zero. Across all quality-controlled 30 min periods (day and night, H QC ≤ 8), 54.7% had β < 0 and 10% had |β| < 0.1, the regime where the (1 + 0.03/β)^−2^ humidity-correction term in the BLS C_t_^2^–C_n_^2^ conversion diverges. Applying CF uniformly to such periods would amplify negative fluxes in a physically inconsistent direction. We therefore compute CF from daytime Rn > 0 periods only and restrict all closure-corrected comparisons to daytime as well. Future studies employing closure correction in late-season or winter datasets should explicitly evaluate the sign distribution of H and β before applying uniform correction factors.

### 4.3. Flux Footprint Representativeness

The probability-weighted composite footprint shows 98% (EC) and 97% (BLS) of the flux source area within the field boundary. The peak of the EC footprint function lies ≈14 m upwind of the tower—about one-quarter of the minimum tower-to-edge distance (56 m)—and the 80% cumulative source area, although more distant, remains within the field, providing a substantial buffer against off-field contamination. The homogeneous peak canopy (mean NDVI = 0.85, range 0.79–0.90) throughout the overlap means footprint contributions are sampled from a single, well-defined phenological state, ruling out intra-footprint vegetation heterogeneity as a confounding factor—a common complication in BLS–EC intercomparisons over mixed or partially senesced surfaces [20,41]. The agreement reported here is therefore specific to peak-canopy, well-irrigated conditions and should not be extrapolated to emergence, rapid-development, or full-senescence stages, nor to other field geometries.

### 4.4. Sensible Heat Flux Comparison

The BLS retrieves a path-averaged C_t_^2^ over 140 m and inverts it through MOST, whereas the EC sonic anemometer is a point sensor measuring from a footprint. Path-averaging and the MOST inversion damp the largest instantaneous H excursions, compressing the BLS dynamic range relative to EC and producing a regression slope below unity even over a homogeneous field [39,40]. This mechanism, rather than the closure deficit, is the most plausible explanation for the ~0.6 slope; the near-zero-β sensitivity of the C_t_^2^–C_n_^2^ conversion documented in Section 3.3 and Section 4.2 adds further uncertainty to H_BLS when |β| < 0.1.

Another reason for the differences is the EC closure deficit. Because the EC system does not close the energy balance, its turbulent fluxes are under-measured by a factor of ~CF (Section 3.3); corrected H would therefore be larger than measured H_EC. This bias measures H_EC low and contributes to the small positive BLS–EC mean difference, together with the documented tendency of scintillometers to read slightly higher H than EC [40,41]. The closure deficit, therefore, helps explain the mean offset but cannot account for the amplitude compression, which is governed by the path-averaging mechanism above.

Importantly, this amplitude offset is not a free-convection approximation artifact. The Scintec Theory Manual [32] establishes that the free-convection solution is only valid when ζ < −0.2 (underestimation exceeds 10% beyond this threshold). The median daytime stability parameter over the overlap was ζ = z_e_^LL^/L ≈ 2.15/(−27) ≈ −0.08, well within the MOST-valid regime. Our processing uses the full iterative MOST framework with the Thiermann and Grassl [44] stability functions and the De Bruin et al. [31] universal temperature function throughout.

A notable feature of the diurnal composite (Figure 8) is a large negative EC excursion during the evening transition (16:00–20:00 LST, reaching −55 W m^−2^) that BLS reproduces only partially (−25 W m^−2^). This difference likely reflects (i) rapid stability changes during the wind reversal from SSW to NE that affect the sonic anemometer’s turbulence statistics, and (ii) advective heat influx that a path-averaged sensor integrates differently from a point-scale sensor.

### 4.5. Latent Heat Flux, Evapotranspiration, and Phenological Control

The contrasting performance between H (systematic amplitude offset) and LE (closer agreement after closure correction) is a well-documented characteristic of residual-based energy-balance approaches [41,45,46]. Because LE = Rn − G − H, errors in all three components accumulate in the residual. Over this well-irrigated peak-canopy field, LE ≈ (Rn − G) and any perturbation in H translates directly into an equal-and-opposite perturbation in LE. Negative nocturnal BLS LE is a fundamental limitation of the residual method when Rn − G is small; ET totals are therefore restricted to daytime (Rn > 0) throughout.

The conditionality of the BLS–EC LE agreement must be stated explicitly: the close agreement after correction is conditional on (a) daytime-only aggregation, (b) shared Rn and G between BLS and corrected-EC LE calculations, and (c) the closure correction itself. Because conditions (b) and (c) are both satisfied by construction, the agreement between BLS residual LE and closure-corrected EC LE reflects internal consistency rather than fully independent validation. Our observed BLS bias against uncorrected EC of +16% falls within the manufacturer-documented 20% expectation, providing another independent line of evidence—alongside the observed closure deficit, the Mauder et al. [12] FLUXNET synthesis (10–30%), and the Twine-corrected comparison (~6%)—that is consistent with the closure-gap interpretation rather than a systematic instrument bias.

Two residual physical processes deserve acknowledgment as additional sources of uncertainty in LE_BLS:

Spatial scale mismatch in Rn and G: The residual LE calculation uses Rn and G measured at the EC tower, which may not represent conditions along the full 140 m BLS path. Deploying replicated Rn and G sensors along the path would allow this mismatch to be quantified directly.

Gap-filling propagation: The large BLS gap fraction (concentrated at night but reaching ~15% during the day) means that a non-trivial fraction of the 30 min LE values entering the daily sums are MDS-filled; gap-filling propagation introduces an “irreducible floor on daily precision.”

### 4.6. Practical Implications for Irrigation Management

For irrigation scheduling, the primary requirement is reliable detection of temporal ET variability. BLS captures this variability effectively: daily ET correlation against uncorrected EC is r = 0.95, and the temporal pattern of ET decline through the senescence transition is reproduced well. After the Bowen-ratio-preserving closure correction, BLS cumulative ET was within −6% of corrected EC (the Mauder correction is similar, at the cost of the non-independence noted above)—a difference within practical irrigation-management tolerances.

Against uncorrected EC, BLS overestimates cumulative ET by +16% (+13.5 mm). For quantitative water-balance applications, this bias would lead to modest overestimation of crop water demand if BLS were used without a correction. A practical approach is to apply a site-specific CF derived from a short concurrent EC deployment, or to treat the BLS estimate as bracketed between uncorrected EC (lower bound) and closure-corrected EC (upper bound), with BLS falling between the two.

These results pertain specifically to a single late-season, well-irrigated potato field in the Beqaa Valley, Lebanon. Broader applicability across MENA requires multi-site, multi-season validation encompassing early-season emergence, summer peak-demand, and deficit-irrigation conditions before generalization is warranted, in line with recent semi-arid Mediterranean ET intercomparison efforts [12,47].

## 5. Limitations and Uncertainty

The following limitations frame the interpretation of results and guide priorities for future work.

**Figure 13:**
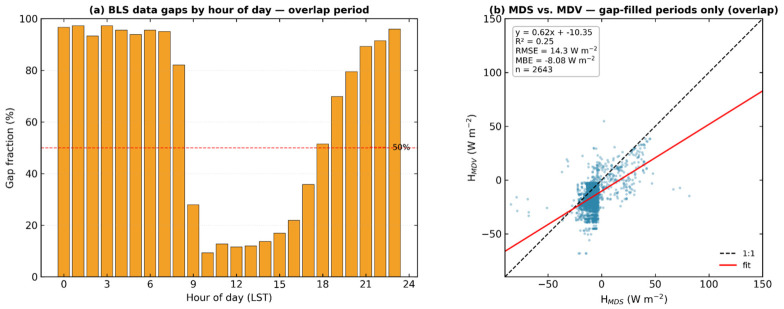
(**a**) Diurnal distribution of BLS data gaps over the BLS–EC overlap period. (**b**) Comparison of MDS and MDV gap-filled H values for the 2643 gap-filled 15 min periods.

First, and most consequential, gap-filling constitutes 61.6% of BLS records over the overlap period (13 October–27 November 2021; 2643 of 4290 fifteen-minute periods) (Figure 13). Core-daytime gaps are low (≈15% over 09:00–16:00 LST, with a minimum near 9% at 10:00) but nocturnal gaps exceed 90%, driven by the scintillometer’s requirement for unstable stratification and sufficient signal strength. The gap-fill uncertainty (MDS vs. MDV RMSE = 14.3 W m^−2^ over the gap-filled overlap periods; the larger RMSE = 18.5 W m^−2^ for the full BLS season, including August–September, reflects the wider range of conditions sampled) is of the same order as the half-hourly BLS–EC RMSE (≈60% of it) and represents the leading source of uncertainty in daily and cumulative H and ET.

Second, negative Bowen ratios are widespread across the quality-controlled periods (54.7% with β < 0), placing the BLS C_t_^2^–C_n_^2^ humidity correction in its most sensitive regime.

Third, energy-balance closure is non-stationary at the sub-seasonal scale: the monthly correction factor declined from CF = 1.26 in October to CF = 1.22 in November (seasonal CF = 1.24). A single fixed factor, therefore, slightly under-corrects the higher-deficit early-season periods and over-corrects later ones, arguing for dynamically tracked closure in future campaigns.

Fourth, the daily scale comparisons rest on a limited number of independent days (*n* = 45 for ET and *n* = 42 for non-gap-filled H), so the daily regression slopes, correlation coefficients, and mean bias errors reported here carry appreciable sampling uncertainty. These metrics should be read as indicators of systematic behavior over a single late-season campaign rather than as precise, transferable population estimates; confirming them would require multi-season deployments spanning a wider range of canopy and stability conditions.

## 6. Conclusions

This study provides the first simultaneous eddy-covariance–scintillometer intercomparison of surface energy fluxes and evapotranspiration over an irrigated crop in the MENA region. Its central contribution is to disentangle two effects that are routinely conflated when scintillometry is benchmarked against eddy covariance. The apparent overestimation of scintillometer-derived ET relative to uncorrected EC is shown not to be an instrument error but a direct expression of the EC energy-balance closure deficit: because the residual method forces closure while the EC reference does not, the two are not measuring the same quantity until the closure gap is reconciled. Once the EC fluxes are closure-corrected, the cumulative ET estimates agree to within ~6%, and the residual day-to-day disagreement falls within the combined measurement and gap-filling uncertainty. This reframing matters beyond the present site, because it implies that scintillometer “biases” reported elsewhere against unclosed EC references may be substantially artefactual.

A second, methodologically distinct finding is that the scintillometer reproduces the temporal structure of sensible heat flux faithfully while compressing its amplitude, yielding a regression slope near 0.60 that is essentially invariant across averaging periods from half-hourly to weekly. This amplitude compression cannot be attributed to the closure deficit—which would act to raise the slope, not lower it—and instead reflects the fundamental difference between a path-averaged, similarity-based retrieval and a point eddy-covariance sensor. The persistence of a near-constant slope under temporal aggregation indicates a systematic rather than random origin, and we verified that it is not an artifact of the free-convection approximation or of stability-regime violations. Separating this scale-invariant amplitude term from the small, closure-driven mean offset clarifies what a scintillometer can and cannot deliver as a stand-alone reference.

The practical implication is that reduced-aperture scintillometry is a credible, lower-cost route to field-scale daytime ET monitoring under well-irrigated, full-canopy conditions, provided its limitations are respected. Chief among these is the high proportion of unretrievable periods—dominated by nocturnal stable stratification—which confines the method to daytime estimation and makes gap-filling, rather than the retrieval itself, the leading uncertainty in cumulative totals. The closure correction central to our interpretation is itself non-stationary at the sub-seasonal scale, arguing against a single fixed correction factor and in favor of dynamically tracked closure. We also caution that the close agreement between residual-method and closure-corrected ET is partly structural, since both draw on the same net radiation and soil heat flux; genuinely independent validation will require spatially distributed available energy measurements along the beam. Because these results derive from a single crop, season, and phenological window, their generalization awaits multi-site, multi-season replication spanning the full-canopy cycle—the priority for subsequent campaigns.

## Figures and Tables

**Table 1:** Instrumentation summary for the EC tower and BLS system.

Instrument	Variable	Height/Depth	System
IRGASON (Campbell Sci., Logan, UT, USA)	3D wind, T_sonic, H_2_O, CO_2_	2 m above the canopy	EC
HygroVUE (Campbell Sci., Logan, UT, USA)	Air T, RH	2.3 m above canopy	EC
CNR4 (Kipp and Zonen, Delft, The Netherlands)	Four-component Rn	2.5 m above canopy	EC
TCAV thermocouples (×3) (Campbell Sci., Logan, UT, USA)	Soil temperature	2, 4, 6 cm	EC
CS655 reflectometers (×3) (Campbell Sci., Logan, UT, USA)	Volumetric water content	3–15 cm	EC
HFP01 soil heat flux plates (×3) (Hukseflux Thermal Sensors, Delft, The Netherlands)	Soil heat flux	8 cm	EC
CR1000X + Granite VOLT 116 (Campbell Sci., Logan, UT, USA)	Data logging	—	EC
BLS900 (Scintec AG, Rottenburg, Germany)	C_n_^2^, C_T^2^	2 m above the canopy	BLS
BLS aperture reducer (Scintec AG, Rottenburg, Germany)	Enables short-path operation	—	BLS
T–RH probe (Scintec AG, Rottenburg, Germany)	Air T, RH	1.8 m	BLS
Two-way pyradiometer (Scintec AG, Rottenburg, Germany)	Rn	2 m	BLS
Wind monitor (R. M. Young Company Traverse City, MI, USA)	WS, WD	2.5 m	BLS
Paired T sensors (Scintec AG, Rottenburg, Germany)	T gradient	1.8 m and 0.3 m	BLS
HFP01 plate (Hukseflux Thermal Sensors, Delft, The Netherlands)	Soil heat flux	8 cm	BLS

**Table 2:** Comparison of BLS ET against EC ET under three closure-correction assumptions (daytime only).

Metric	BLS vs. EC Uncorrected	BLS vs. EC Twine (CF = 1.24)	BLS vs. EC Mauder (Residual → LE)
r	0.95	0.95	0.97
RMSE (mm d^−1^)	0.4	0.35	0.28
MBE (mm d^−1^)	+0.30	−0.14	−0.15
PBIAS (%)	+16.4	−6.2	−6.4
Non-independent	No	No—preferred	Yes—shared Rn, G

## Data Availability

The data presented in this study is available on request from the corresponding author.

## Supplementary Materials

The following supporting information can be downloaded at: https://www.mdpi.com/article/10.3390/s26144398/s1, Figure S1: Study site and potato canopy—Beqaa Valley, Lebanon; Figure S2: Eddy covariance system—full tower view (peak canopy); Figure S3: EC sensor suite close-up and soil sensor installation; Figure S4: BLS900 scintillometer—transmitter, receiver, and meteorological station.
