# Supplementary material for: Eddy Covariance vs. Reduced-Aperture Scintillometry for Potato Crop Evapotranspiration in the Beqaa Valley, Lebanon
Source: Sensors (Basel). 2026 Jul 10;26(14):4398. doi: 10.3390/s26144398 (PMC13417923; doi:10.3390/s26144398)
Supplement: Supplementary file 1 [file sensors-26-04398-s001.zip › sensors-4293758-supplementary.pdf]

Supplementary Material S1: Site and Instrumentation Photographs

Manuscript: ***Eddy Covariance vs. Reduced-Aperture Scintillometry for Potato Crop Evapotranspiration in the Beqaa Valley, Lebanon***

S1.1 Study site and potato canopy — Beqaa Valley, Lebanon

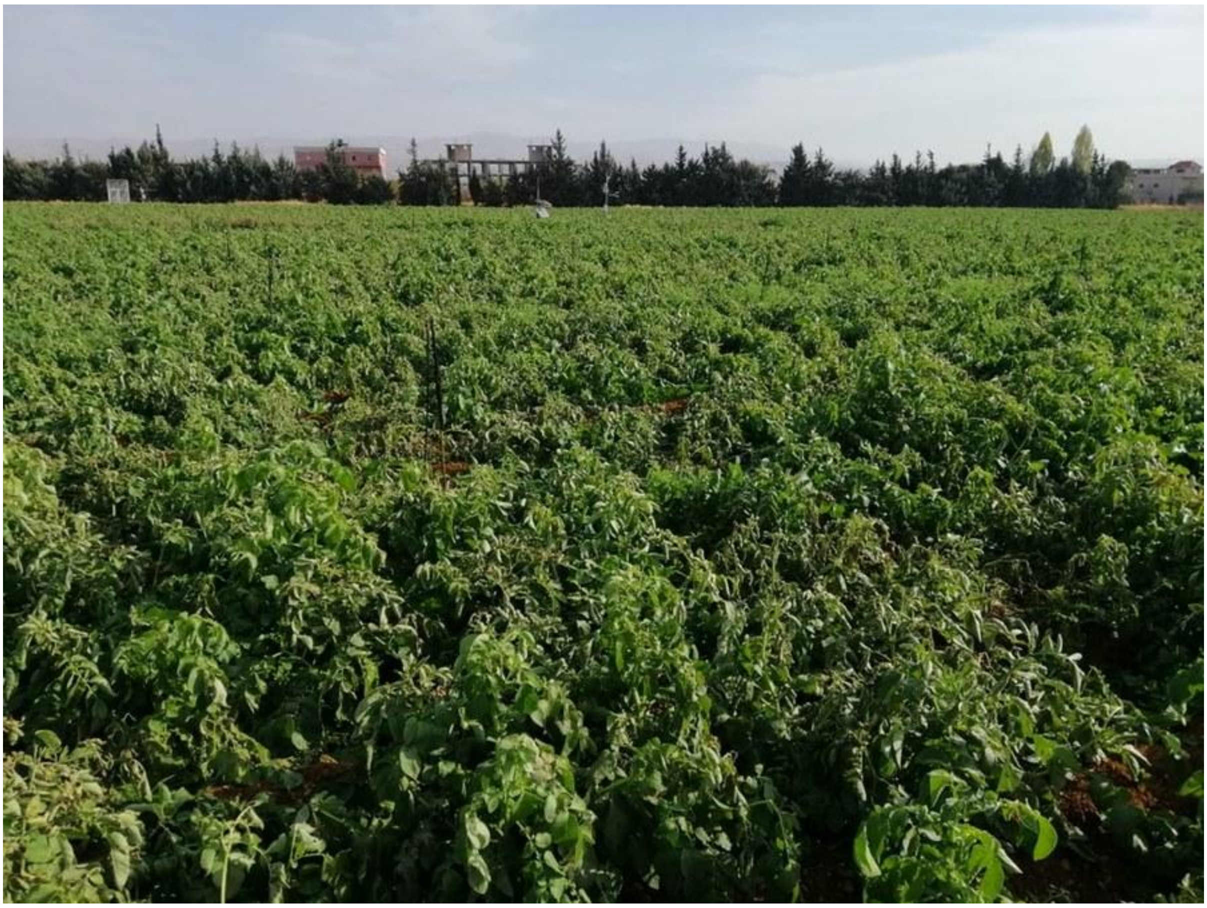

Figure S1. *Potato field (Agria variety, 1.8 ha) at the AREC site, Beqaa Valley, Lebanon (33°55'16.3"N, 36°04'29.0"E; 995 m a.s.l.). The photograph was taken during the BLS–EC intercomparison period (October–November 2021) and shows the late-season canopy at the onset of senescence (NDVI declining from 0.86 toward 0.79) with the EC tower mast visible at mid-field. The AREC facility buildings and the Anti-Lebanon mountain range are visible in the background. The flat, homogeneous topography of the field is consistent with the MOST assumptions underlying both the EC and BLS flux calculations.*

S1.2 Eddy covariance system — full tower view (peak canopy)

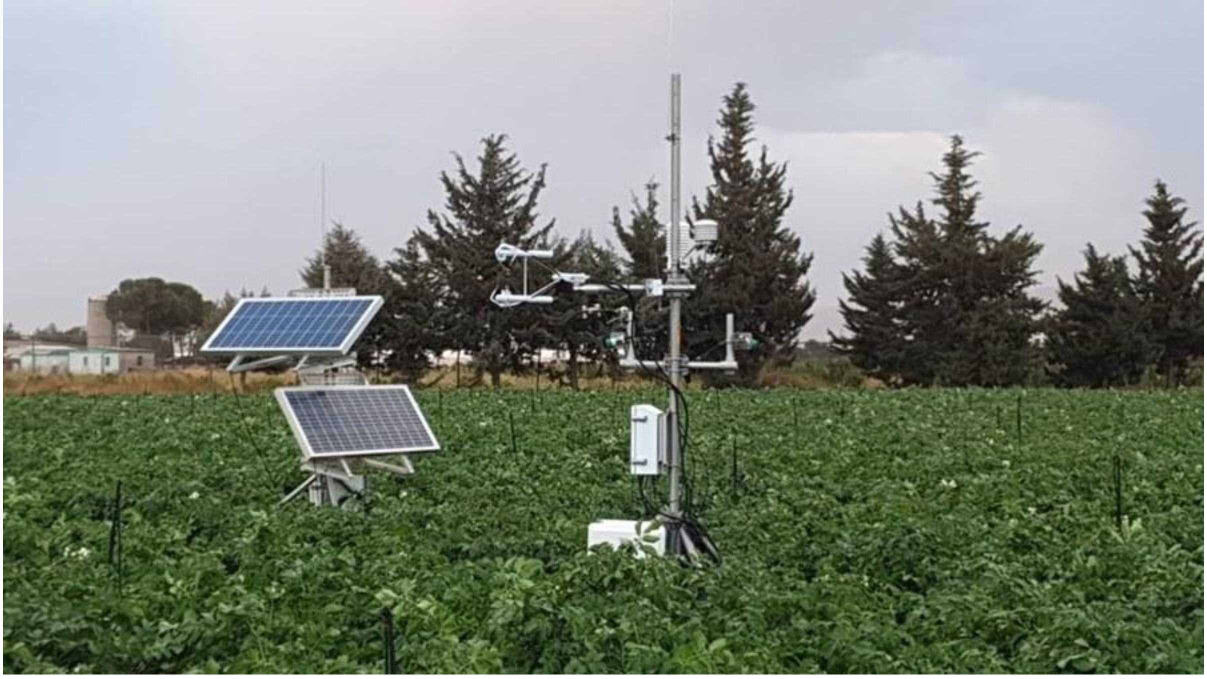

Figure S2. *Eddy covariance tower at peak canopy (Agria potato, canopy height ~0.35–0.5 m, mean NDVI = 0.86; early October 2021). The sensor suite is visible: 3D sonic anemometer and IRGASON open-path CO_2/H_2O analyser at 2 m above the canopy, Kipp & Zonen CNR4 four-component net radiometer, and HygroVUE temperature–humidity sensor. Two solar panels supply continuous power to the CR1000X datalogger (white enclosure, centre). The uniform, dense canopy that characterizes the intercomparison period (13 October – 27 November 2021) is shown.*

S1.3 EC sensor suite close-up and soil sensor installation

(a) EC sensor suite **(b) Soil sensor installation**

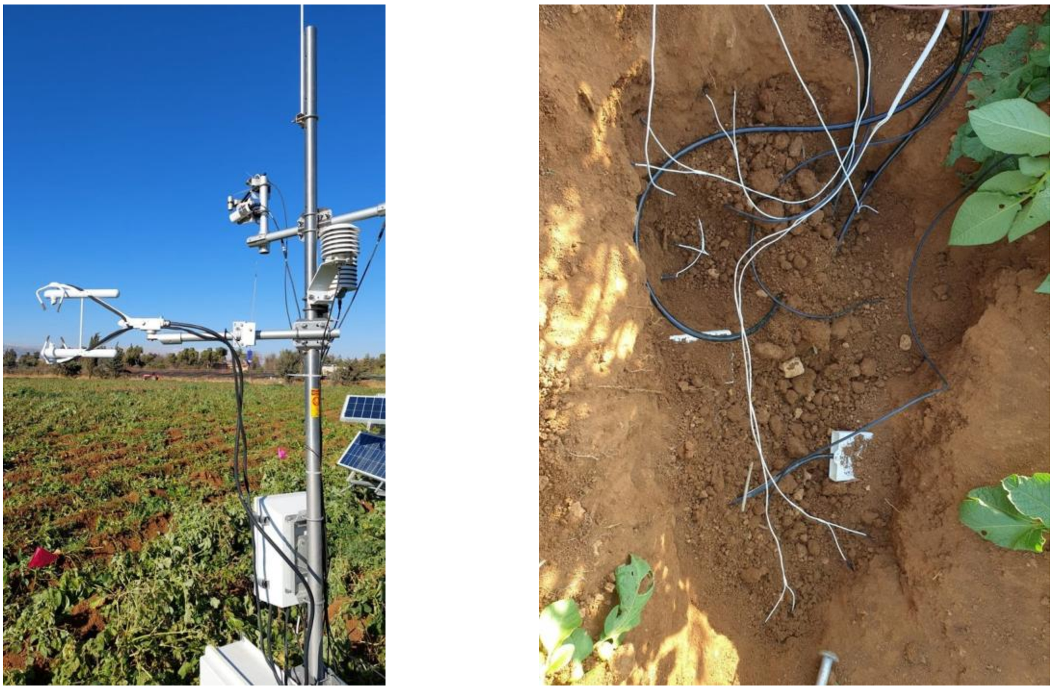

Figure S3. *(a) Close-up of the EC sensor arm showing the Kipp & Zonen CNR4 four-component net radiometer (upper arms, pyranometer and pyrgeometer pairs), Campbell Scientific IRGASON open-path gas analyser at the top of the mast, and HygroVUE temperature–humidity probe (radiation shield, right). The late-season canopy with partial row exposure is visible in the background, consistent with the end of the BLS–EC overlap period (November 2021). (b) Soil sensor installation trench at the EC tower base. Three Hukseflux HFP01 heat flux plates were buried at 8 cm depth, three Campbell Scientific CS655 water content reflectometers at 3–15 cm, and TCAV averaging thermocouple arrays at 2 and 6 cm depth. All sensor cables route to the CR1000X datalogger via conduit.*

S1.4 BLS900 scintillometer — transmitter, receiver, and meteorological station

(a) BLS900 dual-aperture transmitter **(b) BLS900 receiver unit**

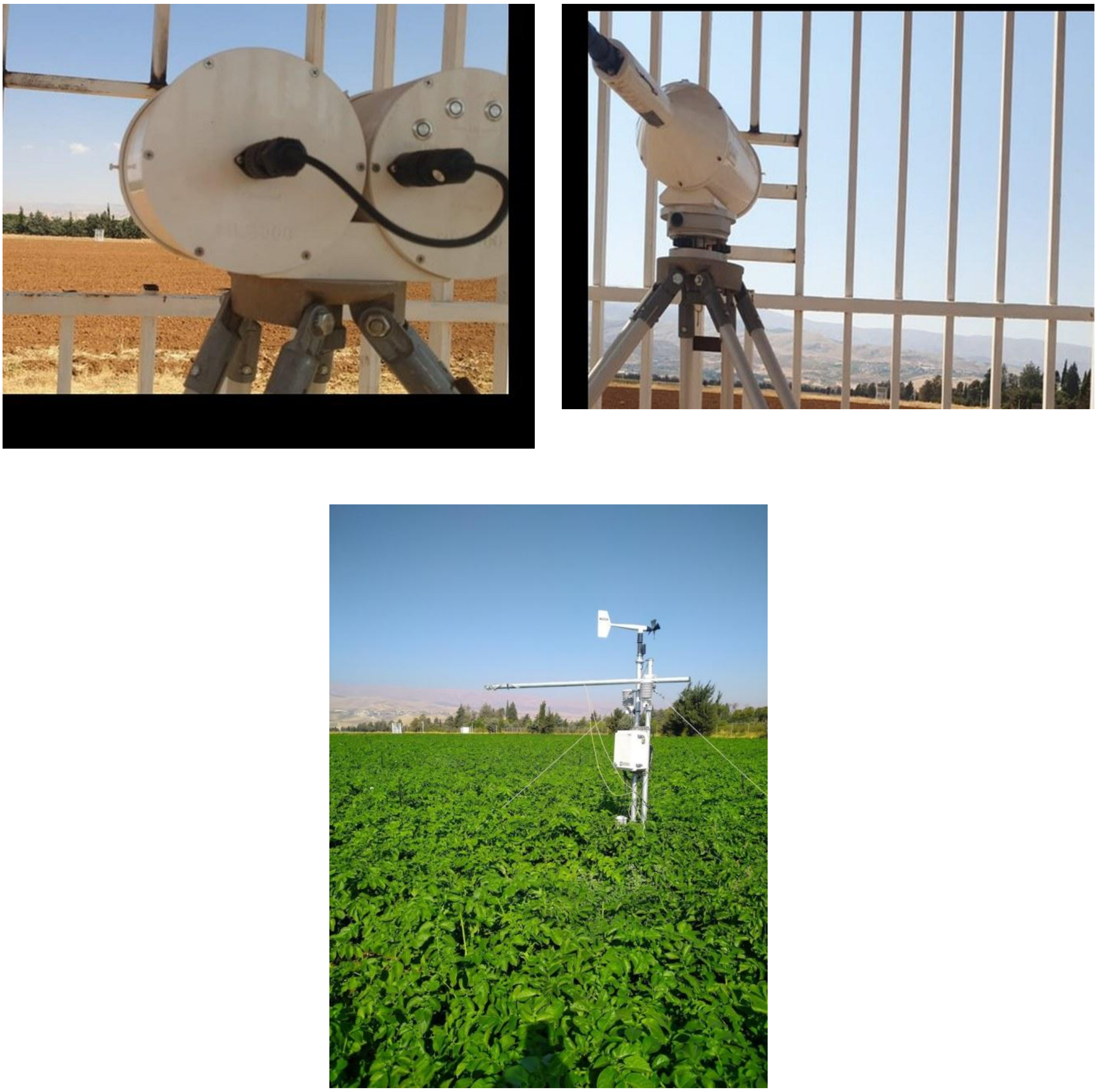

Figure S4. *(a) Scintec BLS900 dual-aperture transmitter (two tangent circular apertures, each D ≈ 0.15 m, visible as the paired white discs). The aperture reducer is fitted to the left (source) disc, reducing the effective emission aperture to enable operation at the short path length of 140 m (standard BLS900 minimum is ~500 m without the reducer). (b) BLS900 receiver unit on adjustable tripod, aligned to the transmitter across the potato field. The Beqaa Valley floor is visible through the receiver housing. (c) BLS meteorological sensor post collocated with the receiver, showing the wind monitor (PropVane), paired temperature sensors (for stability sign determination), temperature–humidity shield, and CR1000X datalogger enclosure, standing in the peak-canopy potato field. The Lebanon mountain range and Beqaa Valley landscape are visible in the background. Path length: 140 m; measurement height: 2 m above canopy.*
